# University-Associated SARS-CoV-2 Omicron BA.2 Infections, Maricopa County, Arizona, USA, 2022

**DOI:** 10.3201/eid2807.220470

**Published:** 2022-07

**Authors:** Nicole Fowle, Brenna Garrett, O’Zandra L. Floyd, Jennifer Collins, Aaron D. Krasnow, Mario Islas, Steven C. Holland, Matthew F. Smith, Efrem S. Lim, Nicole M. Jarrett, Sarah E. Scott

**Affiliations:** Maricopa County Department of Public Health, Phoenix, Arizona, USA (N. Fowle, B. Garrett, O.L. Floyd, J. Collins, N.M. Jarrett, S.E. Scott);; Arizona State University, Tempe, Arizona, USA (A.D. Krasnow, M. Islas, S.C. Holland, M.F. Smith, E.S. Lim)

**Keywords:** COVID-19, respiratory infections, severe acute respiratory syndrome coronavirus 2, SARS-CoV-2, SARS, coronavirus disease, zoonoses, viruses, coronavirus, BA.2 subvariant, Omicron, investigation, epidemiology, disease outbreaks, Arizona, United States

## Abstract

We investigated a university-affiliated cohort of SARS-CoV-2 Omicron BA.2 infections in Arizona, USA. Of 44 cases, 43 were among students; 26 persons were symptomatic, 8 sought medical care, but none were hospitalized. Most (55%) persons had completed a primary vaccine series; 8 received booster vaccines. BA.2 infection was mild in this young cohort.

In November 2021, cases of highly transmissible SARS-CoV-2 B.1.1.529 Omicron BA.1 variant were identified in southern Africa (*1*; F.P. Lyngse et al., unpub. data, https://doi.org/10.1101/2022.01.28.22270044). By January 2022, BA.1 was the dominant variant circulating globally, and the BA.2 variant had been detected in several countries, including the United States (*2*,*3*; F.P. Lyngse et al.). The BA.1 variant causes milder illness compared with the B.1.617.2 and AY (Delta) subvariants, especially in younger persons and vaccinated persons (*4*; J.A. Lewnard et al., unpub. data, https://doi.org/10.1101/2022.01.11.22269045), but clinical severity of BA.2 is not yet well described. We describe illness severity and clinical outcomes of a 44-person US university-affiliated cohort, comprised predominantly of students, who tested positive for BA.2.

On January 24, 2022, the Maricopa County Department of Public Health (MCDPH), Arizona, USA, was notified of a BA.2 cluster in persons at a university. Cases were identified through routine surveillance by the university-affiliated genomics laboratory (Appendix). MCDPH investigated to describe the epidemiologic and clinical outcomes of the cohort. 

We defined a case as a university student or staff member with a SARS-CoV-2 PCR-positive saliva specimen collected during January 3–23 that was tested in the university laboratory and identified as BA.2 by next-generation sequencing. MCDPH and the university distributed electronic questionnaires to all case-patients via text message, email, or both, which is county and university protocol for anyone with SARS-CoV-2 infection (Appendix). MCDPH investigators also conducted telephone interviews with case-patients to collect information on demographics, recent travel, clinical symptoms and outcomes, and vaccination history. We considered a case lost to follow-up if the person could not be contacted by telephone or refused the telephone interview and they did not respond to either electronic questionnaire. We supplemented race/ethnicity (when otherwise unknown), vaccination history, and university clinic visit data by using the Arizona State Immunization Information System and university records.

We defined illness onset as the first date a case-patient experienced any SARS-CoV-2 symptom or the specimen collection date if a person was asymptomatic or lost to follow-up. We categorized vaccination status as unknown or unvaccinated when no documentation of vaccination was available, or a case-patient reported being unvaccinated. We categorized status as completed a primary series when case-patients had documentation of receiving a Food and Drug Administration–authorized or approved vaccination series or a series listed for emergency use by the World Health Organization and considered case-patients boosted when they had documentation of an additional vaccine dose after completing a primary series. We considered a case previously infected if the patient had a SARS-CoV-2–positive PCR or antigen test collected >90 days before BA.2 illness onset in the statewide communicable disease database.

We identified 44 cases, 43 (98%) were in students, which accounted for <1% of 6,268 university-affiliated persons who tested SARS-CoV-2–positive during the study period (*5*). Case-response rate to either questionnaire was 75%. Median age among case-patients was 21 (interquartile range 19–24) years; 29 (66%) were male; 12 (27%) identified as Asian/non-Hispanic, 3 (7%) as White/non-Hispanic, and 29 (66%) as other or unknown race/ethnicity.

At least 26 (59%) case-patients experienced >1 symptom, most of which were consistent with a viral upper respiratory tract infection, such as sore throat, rhinorrhea and cold-like symptoms, cough, and fever (Table). Only 8 (18%) case-patients sought medical attention from the university clinic <7 days before or after their BA.2-positive specimen collection date, but none were hospitalized, and none died.

**Table:** Characteristics of SARS-CoV-2 B.1.1.529 Omicron BA.2 cases among students and staff affiliated with a local university, Maricopa County, Arizona, USA, January 2022*

Characteristics	No. (%)
Median age, y (IQR)	21 (19–24)
Sex	
M	29 (66)
F	15 (34)
Race and ethnicity	
Asian, non-Hispanic	12 (27)
White, non-Hispanic	3 (7)
Other/unknown	29 (66)
University affiliation	
Student	43 (98)
Staff	1 (2)
Case interview response type	
Telephone interview and electronic survey	20 (45)
Electronic survey only	13 (30)
Lost to follow-up	11 (25)
University clinic visit <7 d of illness onset†	
Y	8 (18)
N	36 (82)
Symptom status	
No symptoms	8 (18)
Unknown	10 (23)
Any COVID-19 symptom	26 (59)
Sore throat	18 (41)
Cough	16 (36)
Runny nose, cold-like symptoms	16 (36)
Fever	15 (34)
Muscle aches	11 (25)
Fatigue	10 (23)
Chills	4 (9)
Headache	4 (9)
Shortness of breath	2 (5)
Difficulty breathing	2 (5)
New loss of taste or smell	2 (5)
Diarrhea	2 (5)
Vomiting	1 (2)
Outcome	
Hospitalized	0
Died	0
COVID-19 vaccination status	
Primary series completed, not boosted	24 (55)
mRNA, Pfizer or Moderna	16 (50)
Janssen/Johnson & Johnson	5 (16)
Vaxzevria, Oxford-AstraZeneca	11 (34)
Primary series and booster completed	8 (18)
Unknown or unvaccinated	12 (27)
Median days from primary vaccination series completion to illness onset (IQR)‡	216 (164–269)
Median days from booster vaccine dose to illness onset (IQR)	27 (19–42)

*Illness onset is defined as the first day of symptom onset or the day of positive specimen collection (if asymptomatic or lost to follow-up). IQR, interquartile range. †Within 7 days before or 7 days after illness onset. ‡Excludes case-patients who received a booster dose of COVID-19 vaccine (n = 8).

Of 44 cases, 24 (55%) completed only the primary vaccine series, 8 (18%) received booster vaccines, 12 (27%) had an unknown or unvaccinated status, and 1 (2%) was previously infected with SARS-CoV-2. Of 32 case-patients who completed a primary series, 16 (50%) received an mRNA vaccine, either Comirnaty (Pfizer-BioNTech, https://www.pfizer.com) or Spikevax (Moderna, https://www.moderna.com), 11 (34%) received Vaxzevria (Oxford-AstraZeneca, https://www.astrazeneca.com), and 5 (16%) received Janssen/Johnson & Johnson (https://www.jnj.com).

The mild illness and outcomes we describe might have been driven by the cohort’s age rather than viral characteristics. Because our study involves a university-affiliated cohort, these findings might not be generalizable to more diverse populations. Also, the low telephone interview participation rate prevented collection of close contact information to assess transmission dynamics. In addition, a potential unknown bias in random specimen selection for sequencing could limit the ability to generalize outcomes to this population.

In conclusion, >50% of 44 case-patients in our cohort experienced symptomatic BA.2 infection, but <25% sought medical care, suggesting BA.2 infection in a young population might be mild. In addition, nearly 75% of case-patients completed a primary vaccination series which, in addition to their age, might have contributed to their mild illness. However, data were insufficient to compare if vaccination status affected whether case-patients experienced symptoms or sought medical care. Among persons who completed a primary vaccine series, only 25% received booster vaccines. By March 2022, in alignment with Centers for Disease Control and Prevention recommendations (*6*), >33% of Maricopa County residents >18 years of age had received a booster dose. However, targeted efforts might be needed to encourage booster vaccines among university students (*7*).

Appendix Additional information on SARS-CoV-2 Omicron BA.2 infections among university students and staff, Arizona, USA.
