# Supplementary material for: University-Associated SARS-CoV-2 Omicron BA.2 Infections, Maricopa County, Arizona, USA, 2022
Source: Emerg Infect Dis. 2022 Jul;28(7):1520–2. doi: 10.3201/eid2807.220470 (PMC9239890; doi:10.3201/eid2807.220470)
Supplement: Appendix — Additional information on SARS-CoV-2 Omicron BA.2 infections among university students and staff, Arizona, USA. [file 22-0470-Techapp-s1.pdf]

Article DOI: https://doi.org/10.3201/eid2807.220470

University-Associated SARS-CoV-2 Omicron BA.2 Infections, Arizona, USA, 2022

Appendix

University-Based SARS-CoV-2 Testing

The university has a robust and accessible SARS-CoV-2 testing program in which students and staff are offered testing at any time, regardless of whether they are symptomatic. Students and staff can seek testing without healthcare provider orders. Specimens can be collected by a healthcare provider at multiple campus locations or self-collected specimens can be deposited at multiple easily accessible “drop-off” points. The impetus for testing varies by individual but, anecdotally, is due to the person recently traveling, being symptomatic, or having been notified of exposure.

University PCR testing utilizes the TaqPath COVID-19 Fast PCR Combo Kit 2.0 (Applied Biosystems/Thermo Fisher Scientific, https://www.thermofisher.com) clinical laboratory assay. As part of a baseline genomic surveillance program, specimens were randomly selected for next-generation sequencing (COVIDSeq; Illumina, https://www.illumina.com) from all university-tested PCR-positive specimens. All specimens sequenced as Omicron BA.2 (Pangolin v1.2.124) during the study period were included in this cohort.

The selection of samples to sequence was not biased by any targeted sampling effort. Due to limitations that the Omicron surge provided, not all PCR-positive specimens could be sequenced in real time; however, during the study period, over 40% of all specimens were selected for sequencing. In addition to BA.2, sequences for other variants (such as Delta and BA.1) were also detected in the randomly sequenced specimens within this population during this time.

Electronic Questionnaires

Maricopa County Department of Public Health (MCDPH) electronic questionnaires are sent via text message to all Maricopa County residents with cases of SARS-CoV-2 infection (based on a positive PCR or antigen test) that are reported to the health department with a telephone number. Requested case information in the MCDPH electronic questionnaire includes demographics and living situation (gender, race, and ethnicity; living situation; workplace type); medical comorbidities and COVID-19 vaccination status; illness onset and severity (date of illness onset, if symptomatic; symptoms experienced; whether they required hospitalization or mechanical ventilation); and infection risk factors (prior known contact with someone with SARS-CoV-2 infection; recent travel).

In addition to the MCDPH electronic questionnaire sent to all Maricopa County residents, the university sends a similar electronic questionnaire to university students and staff with SARS-CoV-2 cases. Questions are similar to those in the MCDPH questionnaire, but do not include information about case race/ethnicity, hospitalization status, or workplace type.

Per MCDPH COVID-19 investigations protocol, if a case-patient responded to the MCDPH electronic questionnaire and did not indicate that they live or work in a high-risk or congregate setting (e.g., long-term care facility, correctional facility, etc.), MCDPH and partner investigators would not attempt a telephone interview with the case-patient. In this investigation, MCDPH investigators attempted to contact each university student or staff member with a case of BA.2 infection regardless of whether they responded to the electronic questionnaire. Questions in the telephone interview did not deviate from those in the questionnaire, but phone interviews might have enabled more complete data collection in the case of a person who both responded to the questionnaire and the telephone interview.

Cohort Travel and Previous International Residence

Forty-three cases (98% of total) were identified in university students, of which 10 (23%) reported domestic or international travel, or both, in the 14 days before illness onset. Of those who traveled, 8 (80%) traveled internationally and 7 (70%) reported travel to the same country. Median time from travel return to illness onset was 3 (IQR 3–10) days. Additionally, of student

cases, 36 (84%) resided internationally before enrolling at the university. Of those, 33 (92%) resided in the same country (Appendix Table).

Students who previously traveled or resided internationally accounted for >80% of the cohort, many of whom reported international travel during their exposure period. Travel to countries where Omicron BA.2 subvariant was circulating could explain the association of Omicron BA.2 subvariant infection within this subpopulation. Among those who traveled, infection likely occurred during travel, given median illness onset of 3 days post-travel, which aligns with the Omicron variant incubation period. The high proportion of case-patients reporting travel might be due to increased travel related to Winter holidays. Additionally, most cases occurred in students who previously traveled to or resided in the same international country, which may have increased the likelihood of transmission among shared social contacts.

Appendix Table. Previous international residency status and travel characteristics of SARS-CoV-2 Omicron (B.1.1.529) BA.2–infected students and staff affiliated with a local university, Maricopa County, Arizona, USA, January 2022

Characteristics	No. (%)
University affiliation and previous international residency status	
Student	43 (98)
Previously resided internationally	36 (84)
Previously resided in the country of travel	33 (92)
Travel during 14-d exposure period	
Domestic	2 (5)
International	8 (18)
